# Improving knowledge on the activation of bone marrow fibroblasts in MGUS and MM disease through the automatic extraction of genes via a nonnegative matrix factorization approach on gene expression profiles

**DOI:** 10.1186/s12967-018-1589-1

**Published:** 2018-08-03

**Authors:** Angelina Boccarelli, Flavia Esposito, Mauro Coluccia, Maria Antonia Frassanito, Angelo Vacca, Nicoletta Del Buono

**Affiliations:** 1 Department of Biomedical Science and Human Oncology, University of Bari Medical School, Piazza Giulio Cesare 11, 70124 Bari, Italy; 2 Department of Mathematics, University of Bari Aldo Moro, via Edoardo Orabona, 4, 70125 Bari, Italy; 3 Department of Pharmacology, University of Bari Aldo Moro, via Edoardo Orabona, 4, 70125 Bari, Italy

**Keywords:** Myeloma multiple, MGUS, Fibroblast, Bone marrow microenvironment, NMF, Pathways analysis, Cross-talk

## Abstract

**Background:**

Multiple myeloma (MM) is a cancer of terminally differentiated plasma that is part of a spectrum of blood diseases. The role of the micro-environment is crucial for MM clonal evolution.

**Methods:**

This paper describes the analysis carried out on a limited number of genes automatically extracted by a nonnegative matrix factorization (NMF) based approach from gene expression profiles of bone marrow fibroblasts of patients with monoclonal gammopathy of undetermined significance (MGUS) and MM.

**Results:**

Automatic exploration through NMF, combined with a motivated post-processing procedure and a pathways analysis of extracted genes, allowed to infer that a functional switch is required to lead fibroblasts to acquire pro-tumorigenic activity in the progression of the disease from MGUS to MM.

**Conclusion:**

The extracted biologically relevant genes may be representative of the considered clinical conditions and may contribute to a deeper understanding of tumor behavior.

**Electronic supplementary material:**

The online version of this article (10.1186/s12967-018-1589-1) contains supplementary material, which is available to authorized users.

## Background

Myeloma multiple (MM) is an incurable disease that affects B cells, characterized by the presence of a monoclonal component of plasma cells (PC) in the bone marrow, immunodeficiency, hematopoietic suppression and bone lesions. It accounts for 1% of all cancers and 10% of all haematological malignancies [1]. The growth, infiltration and “homing” of the myeloma cells, as for other cancers, depend on their dynamical interaction with the micro-environment [2]. The bone marrow micro-environment contains a heterogeneous population of cells: hematopoietic stem cells (HSC), stem cells of the bone mesenchymal (BMSCs), vascular endothelial cells and nerve fibres. The BMSCs give rise to a variety of cell types: osteoblasts and osteocytes, adipocytes, chondrocytes and fibroblasts. Certain types of cancers such as adenocarcinoma of the prostate, breast, kidney or lung uses the bone micro-environment as the site of metastasis being this a rich source of growth factors and signaling [3, 4]; on the other hand, for blood cancers, such as MM, the bone marrow is necessary for survival [5]. Indeed, the bone marrow niche appears to play an important role in differentiation, migration, proliferation, survival, and drug resistance of the malignant plasma cells providing the preclinical evidences for targeting MM cells and BMSC as an antitumor strategy in this disease [6]. In the evolution of MM disease, the increase in the monoclonal component of PCs in the bone marrow micro-environment deeply changes homeostasis and interaction with stromal cells. Growth and survival of PCs increases through deregulation of autocrine and paracrine pathways mediated by growth factors, cytokines, angiogenic factors, mRNAs and miRNAs exchanged with exosomes produced by the various stromal components [7–9] and all aberrant stimuli promotes bone destruction [10, 11].

In myeloma study an interesting area of research concerns the understanding of the transformation from precancerous condition [i.e. monoclonal gammopathy of undetermined significance (MGUS)] to a malignant form of the disease. Since MM has a progression of the MGUS clinical condition at a rate of 1% per year of the patients considered [1], the genetic aberrations observed in MGUS are considered to be primary events and once the MGUS clone has been established, immortalization is not enough to promote myeloma progression. In contrast, events in the MM stages that were absent in MGUS are probably secondary events leading to tumor progression [12]. The key questions to be answered in this process are: “why does a clone in MGUS become aggressive in some patients while remaining stable in others”; also “is the different clone behavior dictated by genomic features or is it the result of a plasma cells dialogue and their micro-environment?” The evolving genetic and micro-environmental changes reflect into the progression of the disease. There are concomitant changes in the micro-environment, with a balanced shift between tumor-promoting cells and cancer-suppressing cells, which occur simultaneously with genetic changes in the plasma cell. How these two situations intertwine to mediate myeloma progression is unclear [12].

Studies on the bone marrow stromal component have underlined its important role in the progression of myeloma disease [6, 13, 14]. In solid tumors, it has long been known that in the tumor micro-environment cells are selected as the “activated fibroblasts” (CAFs) [15] that modulate and affect the behaviour of neoplastic cells in order to promote or inhibit growth. These pleiotropic functions highlight the inherent plasticity of fibroblasts; hence learning to the mechanisms that promote them provide new ways to understand and act therapeutically in malignant tumors [16–18]. The CAF is essential in the growth of the primary tumor and in the formation of metastases and it has been observed that in bone marrow it has a role in plasma cell dyscrasias such as MGUS and MM [19–21].

In the light of recent biological studies, the identification of genes potentially involved into the development of MM can facilitate the understanding of the disease etiology and contribute to the advancement of diagnostic tools and clinical research knowledge. However, the automatic extraction of valuable knowledge from microarray data is very challenging since thousands of genes are involved, but only a limited number of samples is available. From a mathematical point of view, this problem is characterized by high data dimensionality. Mathematical methods based on matrix decomposition techniques could be used to explore gene expression data to automatically extract informative patterns to be further investigated from a biological point of view. Particularly, dimensionality reduction methods have many applications in bioinformatics and computational biology since these algorithms act on microarray data reducing the high dimensional gene space (*n*) to a lower dimensional ($r<<n$) gene component space, which is representative of some latent information embedded into the original data [22, 23]. Moreover, dimensionality reduction mechanisms can be used as the first step in classification procedure to help in extracting attribute or dimension which are considered highly relevant with respect to a given class [24–26]. Nonnegative matrix factorizations (NMFs) are data reduction and exploration algorithms which emerge in literature panorama as useful tools for analysing gene expression data because of their inherently non-negativity property [27–30]. NMF methods exhibit a number of properties, being able to (i) find sets of genes co-operating in a relatively tightly regulated manner [31]; (ii) recognize potential relationships in large biological data samples and link genes to these patterns [28, 32]; (iii) uncover distinct genomic subtypes in cancer patients [33]. Differently from classical techniques for dimensionality reduction, such as PCA or SVD, which contain both positive and negative values in the decomposed factor matrices, NMF is able to decompose data matrices with factors only containing non-negative values, representing in this way the original data by only additive, not subtractive, combinations of the basis vectors (metagenes). This parts-based representation of NMF is appealing because it reflects the intuitive notion of combining parts to form a whole and could better uncover meaningful biological interpretation of data matrix [27, 28, 34, 35]. In this study, we developed a NMF-based approach to mine the genetic expression of fibroblasts of patients with MGUS and MM for automatically extracting genes that can be associated with the activation of bone marrow fibroblasts in MM patients. The NMF-based extraction method together with an ad hoc designed post-processing procedure allowed to extract from a large set of fibroblast genes very few genes which underwent to biological functional analysis. The interpretable knowledge obtained thanks to the synergic use of mathematical and biological data analysis complement existing biological hypothesis on a certain influence of fibroblasts that have acquired tumorigenic properties in the progression of the disease from MGUS to MM.

## Methods

### Patients and samples processing

Eighteen patients fulfilling the International Myeloma Working Group diagnostic criteria for MM (n = 10) and MGUS (n = 8) were studied at diagnosis [1]. The MM patients (8 male, 2 female) were staged as IIA (n = 3), IIIA (n = 7); the M-component was Ig G (n = 5), Ig A (n = 2), and k (n = 8) or $\lambda$ (n = 2) [36]. The MGUS patients (6 male, 2 female) were Ig G (n = 6) or Ig A (n = 2). The study was approved by the local ethics committee of the University of Bari Medical School, Italy, and all patients gave their informed consent in accordance with the Declaration of Helsinki (https://www.wma.net). Fibroblasts isolated from each of 18 patients were distinctly used in all experiments [20]. Table 1 summarizes clinical parameters of the bone marrow donors. Briefly, bone marrow aspirates were centrifuged on ficoll-Hypaque gradient centrifugation, and the separated mono-nuclear cells were left to adhere to 25-cm^2^ polystyrene flasks in complete medium (RPMI-1640 medium supplemented with 10% fetal calf serum (FCS) and 1% glutamine) for 24 h in culture conditions.

**Table 1:** Clinical parameters of the bone marrow donors, the categories based on the International Myeloma Working Group uniform response criteria

Case	Sex	^a^IgIsotype stage	
1-MM	F	IgA k	II A
2-MM	M	IgG k	II A
3-MM	M	IgM k/IgA k	II A
4-MM	M	IgG $\lambda$	III A
5-MM	M	IgG k	III A
6-MM	M	IgG k	III A
7-MM	M	micromolecular k	III A
8-MM	F	IgG k	III A
9-MM	M	IgA $\lambda$	III A
10-MM	M	micromolecular k	III A
1-MGUS	F	IgG $\lambda$	
2-MGUS	M	IgG $\lambda$	
3-MGUS	M	IgG $\lambda$	
4-MGUS	M	IgG k	
5-MGUS	M	IgG k	
6-MGUS	M	IgA k	
7-MGUS	M	IgA k	
8-MGUS	F	IgG $\lambda$ / IgA $\lambda$	

^a^The phenotype was investigated with immune-cytochemical staining with anti-k or anti-$\lambda$ antibody according to the light chain of the M-component

Adherent cells were stromal cells were harvested in trypsin/ethylenediaminetetraacetate (EDTA) solution (0.05/0.02% in phosphate-buffered saline, [PBS]), washed twice with PBS, suspended in FCS-free medium (SFM), and immune-depleted of macrophages and possible residual plasma cells by a 30-min incubation in CD14 (a monocyte-macrophage marker) plus CD38 (a plasma cell and hematopoietic cell marker) monoclonal antibody (MoAb) coated flasks (Immuno-tech, Coulter). The fibroblasts were separated using anti-fibroblast micro-beads and their positive fraction wss collected bone marrow fibroblasts purified are grown in 75 cm^2^ flask at 3 °C, 5% CO_2_ in DMEM containing 10% fetal calf serum (FCS), 100 U/ml penicillin and streptomicin (Euroclone UK) and they were used within a 12-h interval; that is, only from the samples that, thanks to the number of fibroblasts, reached 80% confluence for RNA extraction.

### RNA isolation and label protocol

Total RNA was extracted following the standard Trizol protocol (Thermo fisher Scientific). RNA quantification and quality control was performed by Experion RNA STN-SENS Analysis on EXPERION automated electrophoresis station (Bio-Rad Laboratories). Aliquot of total RNA (1 μg) was retro-transcribed and labelled using the Amino Allyl MessageAmp® II aRNA Amplification Kit (Ambion) according to manufacturer’s protocol. Before hybridization, the Cy3 and Cy5 (GE Healthcare-Amersham) labelled samples were combined and dried in a speed vac. To the dried sample 330 μl of hybridization buffer were added. The samples were denatured for 5 min at 65 °C, snap cooled on ice for 1 min. The solution was pipetted onto the microarray MICROMAX glass slide SuperChip I (Cat No. MPS696) provided by PerkinElmer Life Sciences Inc, placed in a hybridization chamber and the cover slip was placed carefully. Hybridization reaction was performed overnight in a sealed chamber (Corning® hybridization chambers, Sigma) at 42 °C in a high-precision Techne Hybridizer Oven HB-1D (Barloworld Scientific Techne). Pre and post-hybridization washing were performed according to the protocol described in Molecular Cloning a Laboratory Manual [37].

### Scan protocol and data processing

Fluorescence signals were detected by analysing the microarrays in a VersArray ChipReader® 5 μm dual confocal laser scanner, with VersArray ChipReader v3.1 software (Bio-Rad Life Sciences Division); for each microarray slide, two images were produced by illuminating the array at 635 nm (excitation of Cy5) and 532 nm (Cy3). For both illuminations, photomultiplier tube (gain and light amplification) settings were at 1000 and laser power was set at 50%. All images were captured in TIFF format. Raw images were analysed with VersArray Analyzer Software v4.5 (Bio-Rad Laboratories) using media pixel intensities for each spot. The set consists of 21,329 oligonucleotides whose length is 70mer (version Operon 2.0) designed on Cluster of Human Unigen, mainly in the 3 end terminal region. Global background was subtracted by bi-quadratic polynomial approximation, and cross-channel normalization was performed by local regression (LOESS).

### Data approximation via nonnegative matrix factorization

**Fig. 1:**
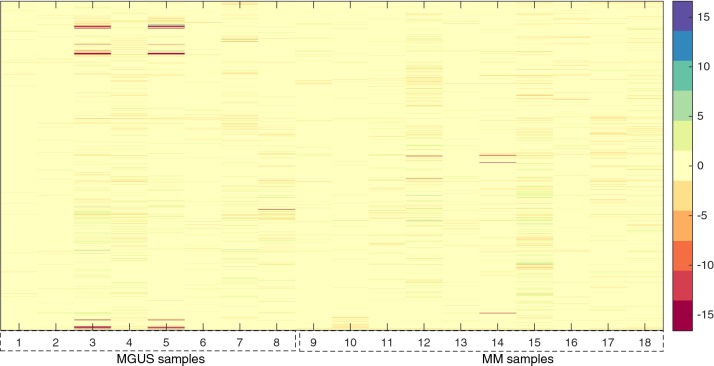
Concatenation of the two conditions: MGUS and MM. Heatmap plot of the concatenated data matrix in log-2 scale: the first 8 columns of the matrix represents the gene expression profile from MGUS conditions while the latter 10 columns represents the gene expression profile from MM conditions

The gene expression profile obtained by the biological experiments are collected in numerical nonnegative matrices whose columns measure the processed intensities of one gene probe in a corresponding experiment on a single patient and rows correspond to the processed intensity for a single gene probe across all the patients. Three different microarray matrices were considered: the first one collects gene expression profile from 8 patients affected by MGUS condition, the second one collects gene expression profile from 10 patients affected by MM and the third one resulting from a concatenation of the previous two matrices. This latter describes gene expression profile among all 18 patients with both clinical conditions (8 with MGUS and 10 with MM). These data matrices have been deposited in the NCBI Gene Expression Omnibus [38] and are accessible through the series entry GSE24990^1^; while the id of the adopted genes are available using the platform entry GPL2136^2^ (totally we considered 21520 genes involved in the analysis). Figure 1 illustrates the heatmap plot^3^ of the concatenated data matrix in log-2 scale: the first 8 columns of the matrix represents the gene expression profile from MGUS conditions while the latter 10 columns represents the gene expression profile from MM conditions.

The three microarray matrices were independently analysed using a nonnegative matrix factorization (NMF) algorithm based on Kullback–Leibler divergence^4^ with multiple runs and random initialization [32, 40]. Numerical experiments were conducted using NMF package on R-project environment [41].

A NMF algorithm approximates any microarray matrix $X\in \mathbb {R}^{n\times m}$ as the product of a nonnegative basis matrix $W\in \mathbb {R}_{+}^{n\times r}$ and a nonnegative coefficient matrix $H\in \mathbb {R}_+^{r\times m}$, so that $X\approx WH$. NMF reduces the dimensionality of available microarray matrix and extracts from it a small number (*r*) of nonnegative features (basis vector) which are indicative of latent knowledge embedded in data [35, 42]. In this paper, the number *r* of metagenes is empirically chosen as described in [40].

Considering gene expression levels from a single sample as a vector in the space of the *n* genes, a column $X_{:,j}$ of the matrix *X* can be interpreted as a nonnegative linear combination of the columns $W_{:,k}$ of the basis matrix *W*, weighted with coefficients of the matrix *H*, that is $X_{:,j} \; \approx \; \sum _{k=1}^r{W_{:,k}H_{kj}},$ for $j=1,\dots ,m.$

**Fig. 2:**
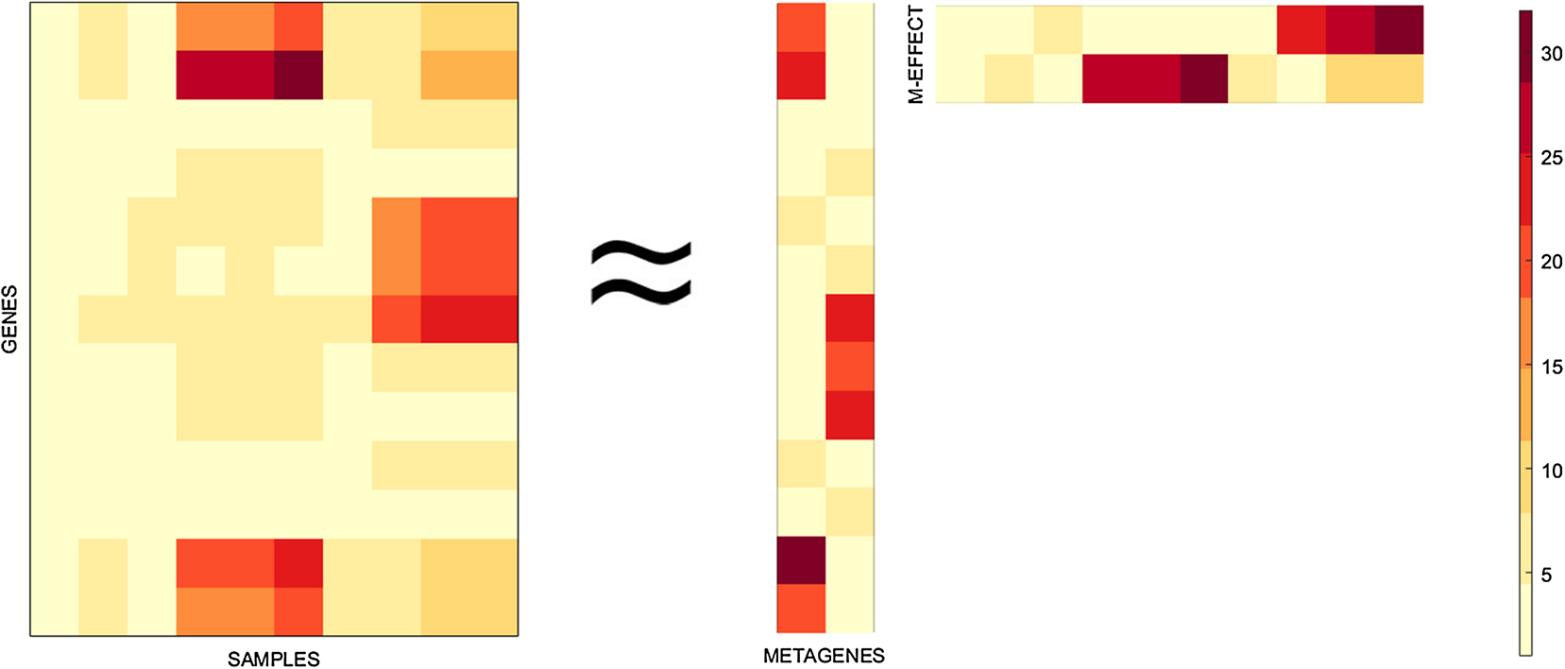
Graphical illustration of NMF. Microarray matrix *X* is modeled as the linear combination of a set of patterns, the columns of *W*, and the assignment of genes to those patterns with varying strengths, the rows of *H*

For the sake of illustration, Fig. 2 reports the heatmap plot of a rank 2 factorization ($r=2$) of a microarray matrix *X* approximated as the matrix product *WH*. Each value in the matrices *X*, *W* and *H* correspond to a color in accordance to the corresponding color bar, rows in *W* represent the same genes in *X*, the two columns of *W* are the 2 metagenes which store up the biological information hidden in the analysed genes, while elements of *H* measure the effect of a specific metagene in a particular sample in *X*.

### NMF based extraction approach and post-processing phase

After the two factor matrices *W* and *H* have been obtained via a NMF algorithm, a procedure to identify relevant genes in each metagene is applied. Particularly, genes in each metagene are firstly ranked and successively extracted in accordance to the some criteria (preliminary chosen). In this work, the *gene*.*score* procedure proposed in [43] is adopted. This procedure selects representative genes in a single metagene if their *gene.score* values are higher than $\hat{\mu }+3\hat{\sigma }$ (where $\hat{\mu }$ and $\hat{\sigma }$ are the median and the median absolute deviation of *gene.scores*, respectively) and their maximal values in the corresponding rows of *W* is larger than the median of all elements in the basis matrix *W*.

In this paper, each of the three microarray data matrices was decomposed via NMF factorization, and then a single metagene was considered for each obtained basis matrix. The single metagene was chosen as the column in the basis matrix possessing the largest number of genes extracted when the gene extraction technique is adopted. Genes extracted from the “most informative” metagenes of the MGUS, MM and the concatenated matrix were subsequently collected into three subsets, indicated hereafter as metaMGUS, metaMM and metaMGUSMM.

With the aim of investigating the influence of specific genes on the disease behavior a post-processing phase was adopted. Post-processing procedure consists in inspecting common and uncommon genes between metaMGUS and metaMM, in associating them the correspondent gene symbol identification and in discarding obsolete genes contained in the Operon version 2.0 platform.

### Considerations on the NMF-based approach

Nonnegative matrix factorization is the core of the peculiar approach we used to extract few genes which should be representative of the whole dataset. NMF was used to performed a dimensionality reduction of data matrices. NMF are applied in a new peculiar way: it is used to select a single metagene from the MGUS data matrix and from the MM data matrix which can be interpreted as the most representative of the whole dataset (for each data matrix). Then, genes in each metagenes were firstly ranked and successively extracted in accordance to the gene score procedure based on the work in [43]. Finally, intersection and complementary set operations has been applied to extract common and uncommon genes to be further investigated from a biologically point of view (details are reported in “Function analysis” section).

It should be observed that factor matrices derived from the NMF decomposition only contain non-negative values, hence the original data can be represented by only additive, not subtractive, combinations of the basis vectors (metagenes). This parts-based representation of original data is appealing because reflects the intuitive notion of combining parts to form a whole. Particularly, the metagenes can uncover meaningful biological interpretation in term of genes it is composed by. Furthermore, each original sample is represented by metagenes with the corresponding encoding vector (the column of coefficient matrix *H*). It is clear that large value of both basis factors and encoding vectors play important role in representing of the original data allowing an intuitive ranking of the most important information.

## Results

### Preliminary microarray data analysis

**Fig. 3:**
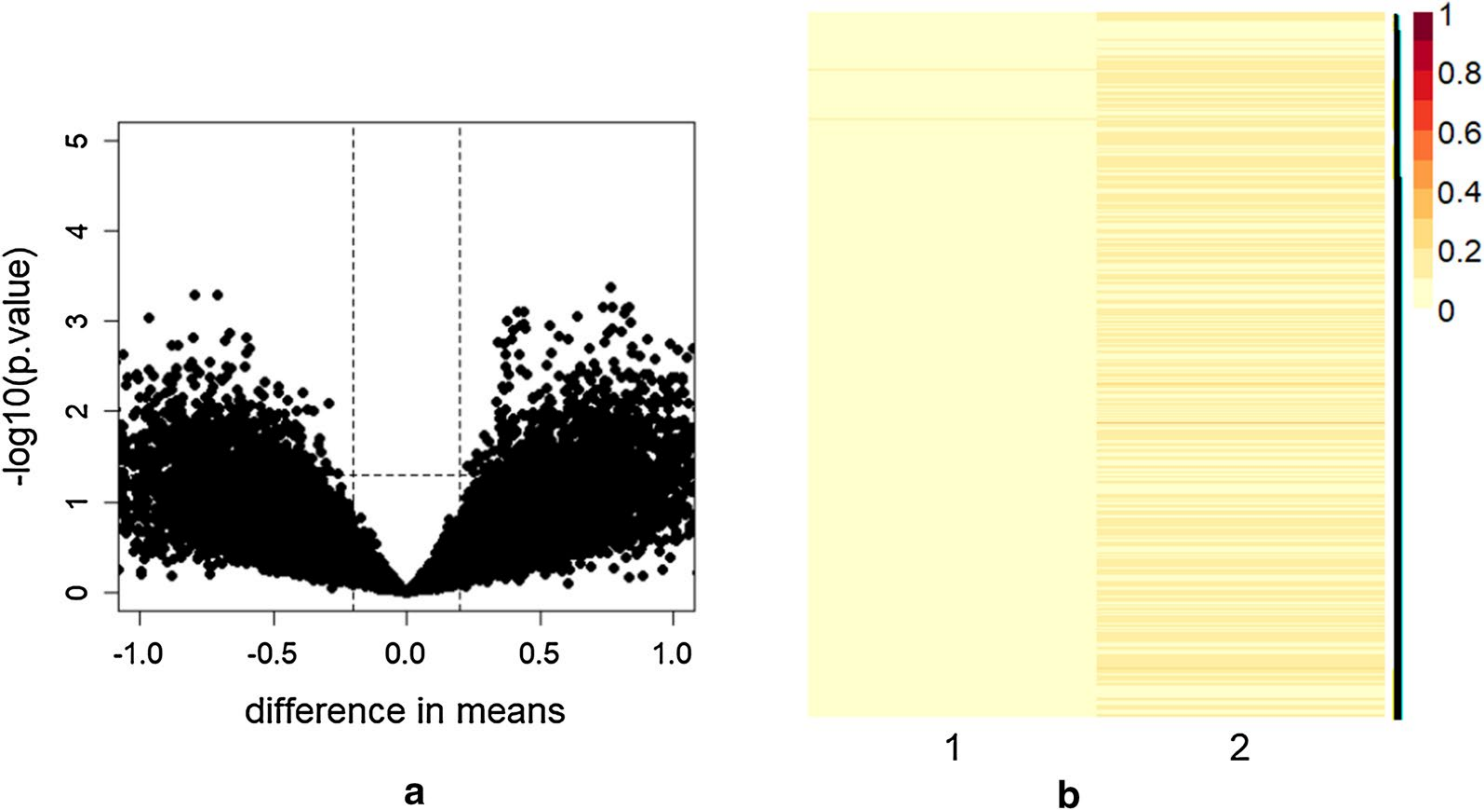
**a** Volcano plot of the gene expression profile data of all 18 patients. *x*-axis reports difference of the group means while *y*-axis indicates statistical significance of the t-test per rows (–$\log _{10}$ of *p*-value). The dashed line shows where $p = 0.05$ with points above the line having $p < 0.05.$ In particular, points represent interesting genes, in the left upper corner are depicted genes with mostly small *p*-value and low difference in means, whereas in the right upper corner there are genes with small *p*-value and large difference in means. **b** Heatmap of the first principal component PC1 and of the Metagene 1 (both normalized) obtained respectively by PCA and NMF on the gene expression profile data of all 18 patients

A preliminary qualitative investigation of the original data matrices were performed to figure up the most appropriate dimensionality reduction mechanism to be applied. Figure 3a illustrates the Volcano plot of the elements in the gene expression profile matrix among all 18 patients with both clinical conditions MGUS and MM. It can be observed the presence of some interesting genes which differentiate in their expression (points above the horizontal dotted line). To obtain information on the overall structure of the complete gene expression microarray data (illustrated in Fig. 1) both principal components analysis (PCA) and NMF were applied. Figure 3b reports the heatmap of the first principal components (PC1) and of the relavant metagene obtained, respectively, using PCA and NMF of the gene expression profile matrix among all 18 patients. As it can be observed, the first principal component, which preserves mostly the variance of data (in particular the 99%), highlights very few genes which are unlikely assumed as a subset of genes representative of the whole dataset. On the contrary, the first metagene obtained using NMF presents differences between genes. Similar plots can be obtained for all the other principal components. These results suggest that PCA fails to detect relevant embedded information while NMF is more effective providing a more comprehensive detection of underlying genetic information of the complete gene expression data and the possibility to handle the results as significance factors [32].

### Microarray dimensionality reduction

**Fig. 4:**
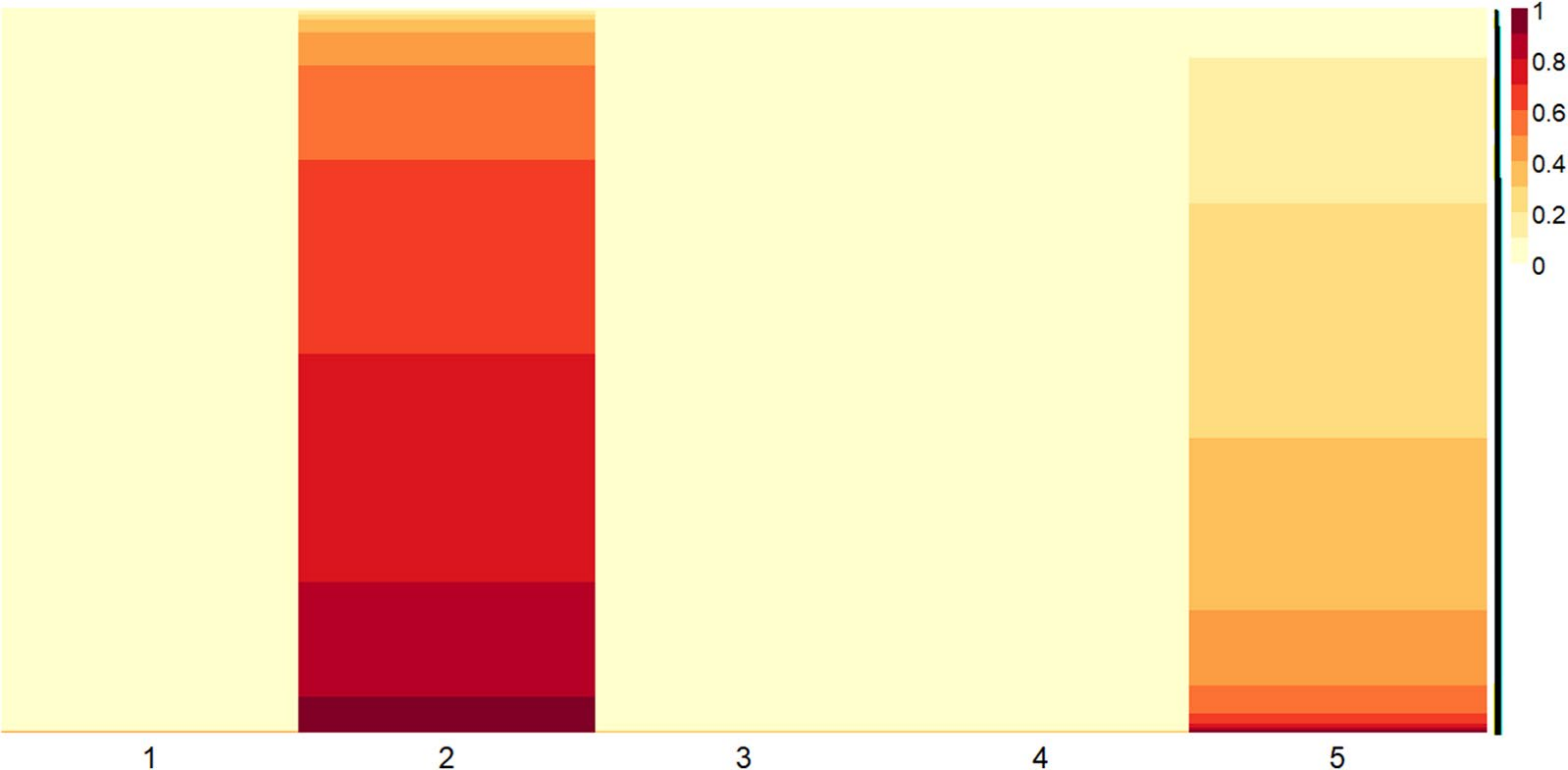
Basis matrix of MGUS condition. Heatmap plot of basis matrix extracted from the gene expression profile data matrix of the 8 patients with MGUS conditions. Five metagenes were automatically extracted with a rank of the factorization = 5. Values in each column of the basis matrix have been normalized by row and sorted to show higher values on the bottom and lower values on the top of the heatmap. As highlighted by the color shades, both metagenes two and five present a significant number of important genes. Metagene two has been considered as the most representative of the whole dataset of MGUS condition since this column includes the largest number of extracted genes

**Fig. 5:**
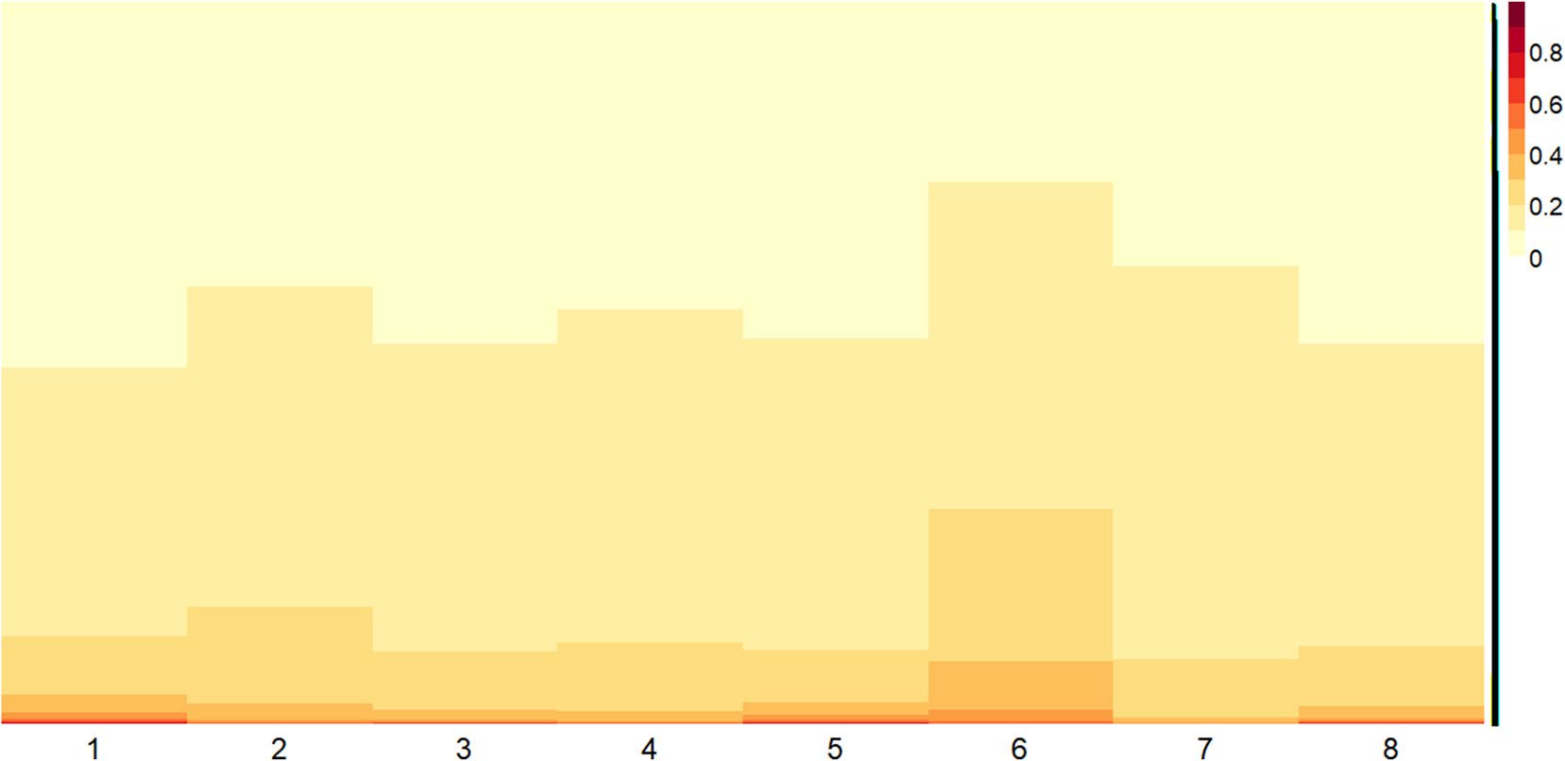
Basis matrix of MM condition. Heatmap plot of the basis matrix extracted from the gene expression profile data matrix of the 10 patients with MM conditions. Eight metagenes were automatically extracted with a rank of the factorization equal to 8. Values in each column of the basis matrix have been normalized by row and sorted to show higher values on the bottom and lower values on the top of the heatmap. Due to the presented of the highest number of relevant values, metagene six was (automatically) identified as the most representative metagene of the dataset with MM condition

The results automatically obtained from NMF based approach previously described are illustrated using heatmap plots. Particularly, Figs. 4 and 5 report the heatmaps of reordered basis matrices extracted from gene expression profiles of 8 patients with MGUS condition and from 10 patients with MM condition, respectively. The ascending re-ordering of each column in the heatmaps helps to identify the relevant metagenes as those with much darker shades than the others. From these metagenes, the gene extraction procedure is performed to automatically extract genes with higher values. These genes constitute the knowledge base to be further investigated from a biological point of view. The extracted subsets: metaMGUS and metaMM composed by 2086 genes and 472 genes, belong to the second column in Fig. 4 and to the sixth metagene in Fig. 5, respectively.

**Fig. 6:**
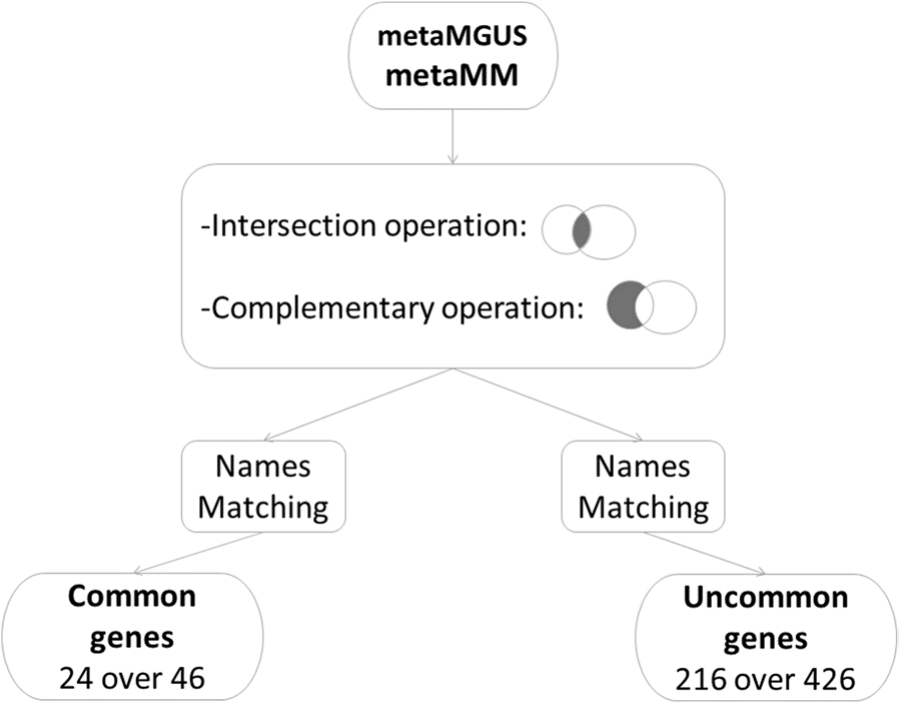
Workflow of the post-processing procedure. Common and uncommon genes have been extracted from the two obtained subsets and matched with their corresponding gene symbols. These operations get two groups of genes: 24 over 46 for common genes and 216 over 426 for uncommon genes. Genes symbols and their corresponding expression median value in the two conditions, MM and MGUS, are reported in Additional file 1

The post-processing procedure is detailed in Fig. 6. As it can be observed, common genes between metaMGUS and metaMM, with the gene symbol identification were only 24 among 46, while the remaining 17 were Homo sapiens cDNA and 5 Hypothetical protein. On the other hand, from the 426 uncommon genes belonging only to metaMM, 226 had the gene symbol identification, while the remaining 136 genes were Homo sapiens cDNA, 35 were hypothetical protein and 29 were not Homo sapiens. Moreover, from 226 genes only 216 were considered useful for the functional analysis since the presence of some duplicates. These genes and their corresponding median value of MGUS and MM are listed in Additional file 1: Tables S1 and S2.

### Function analysis

Common and uncommon genes found in the two metaMGUS and metaMM subsets could be considered as genes potentially involved in the activation of fibroblasts from the MGUS to MM condition.

These automatically selected genes undertook functional analysis via WebGestalt (WEB-based GEne SeT AnaLysis Toolkit) tool^5^ to understand if any genes could be responsible for the activation of bone marrow fibroblasts [44].

The identification of a common genes subset between the fibroblasts of patients with MGUS and the myeloma clinical condition shows that a functional switch is required to lead fibroblasts to acquire pro-tumorigenic activity. This functional switch is determined by genes that promote a phenotype capable of creating a more important dialogue in the bone marrow micro-environment. Results of this functional analysis are described in the following.

#### Functional analysis of common genes

The analysis of the 24 common genes between metaMGUS and metaMM allows to identify four genes in different pathways: 1 prokineticin precursor (Prok-1), gonadotropin releasing hormone 1 leutinizing-releasing hormone (GNRHR), alpha-2-HS-glycoprotein (AHSG) and beta-2-microglobulin ($\beta$2-M). These genes alone may not be related to the fibroblast activation process, however, it is possible that integrated into the functional activity of the uncommon metaMGUS and metaMM subsets these genes are able to work in concert and favour activation. It can be noted that

- The Prok-1 and GNRHR genes are present in the pathways: GPCR ligand binding, signal transduction and class A/1 (rhodopsin-like receptor).
- The AHSG and B2M genes are present in VEGF and VEGFR signaling network, Arf6 downstream pathway, Sphingosine 1-phosphate (S1P) pathway, proteoglycan syndecan-mediated signaling events, Nectin adhesion pathway, EGFR-dependent signaling events endothelin, and endothelins.

In particular, the gene Prok-1 is involved in the synthesis of a secretory protein signalling and is a potent angiogenic factor, in fact, promotes angiogenesis in various steroids glands [45]. Moreover, Prok-1 protein promotes the survival and differentiation of granulocytes and monocytes, as well as stimulation, mobilization of hematopoietic cells and modulation of the immune response [46].

The protein encoded by the gene is a GnRH receptor type 1 of gonadotropin-releasing hormone, it is part of a system autocrine regulation of cell proliferation and it is expressed on the surface of many cells, on lymphocytes and in various human malignant tumors [47].

Alpha2 HS-glycoprotein is encoded by AHSG gene, it is an important chemoattractant in serum or blood and it is involved in different functions as endocytosis and bone formation. Cancer cells have the ability to follow the concentration gradient of Alpha2 HS-glycoprotein from primary sites up to the nearby blood vessel. Moreover, AHSG shows synergy with traditional chemotactic as SDF-1/CXCL12 to mediate chemotaxis and invasion of cancer cells through the extracellular matrix [48].

Finally, the $\beta$2-M gene encodes for a non-glycosylated protein that is present in all nucleated cells. The $\beta$2-M protein activates pleiotropic signaling such as regulation of protein kinase A, androgen receptor, VEGF, fatty acid synthetase and has multiple roles in tumorigenesis and angiogenesis. $\beta$2-M behaves as a growth factor and is known to activate stromal cells, such as mesenchymal stem cells, osteoblasts and osteoclasts [49].

In addition to the described genes, we can individually identify WHSC1, PRDM14 and ANXA11 genes which are involved in autoimmune disorders and in some cancers [50–52]. The set of functions governed by the genes selected and described has shown a particularly active state of the fibroblasts belonging to the subset of common metaMGUS and metaMM genes but without the adoption of a phenotype that can alter the bone marrow.

#### Functional analysis of uncommon genes

**Fig. 7:**
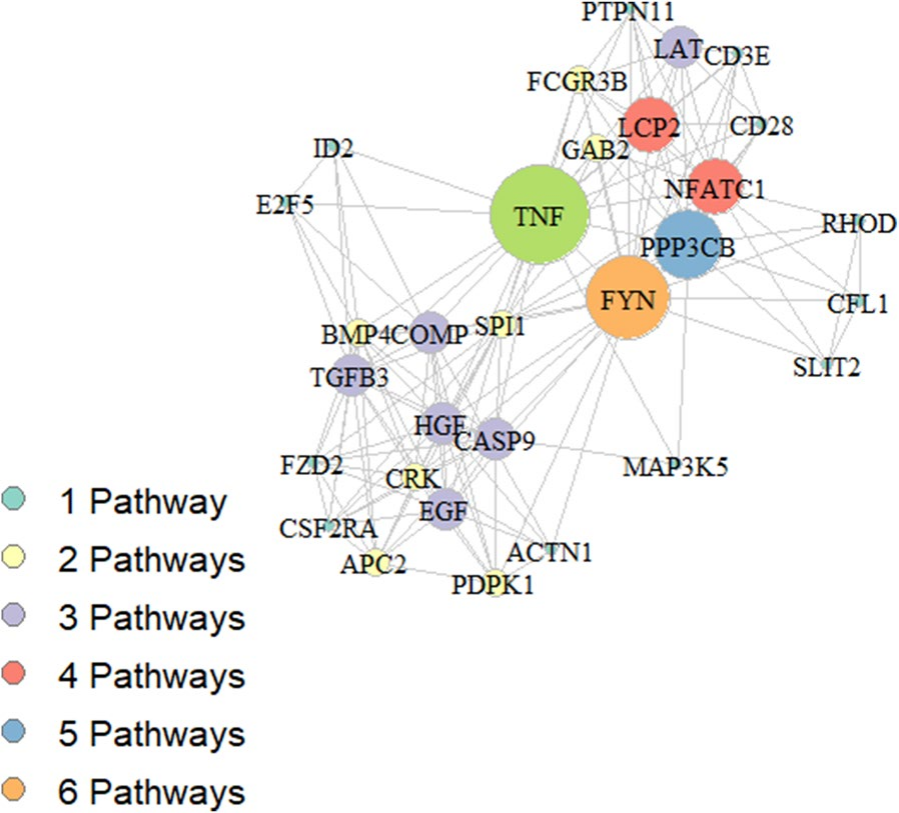
Network obtained from genes sharing different pathways. Nodes of the graph represent the 30 genes reported in Table 2, the graph edges link genes belonging to the same pathway. Node size reflects the number of pathways the gene is involved in: larger is the radius of the node greater is the number of pathways the gene belongs to

The analysis of 216 genes present just in metaMM but not in the intersection allows to select 11 pathways in which only 30 of 216 genes belonging to metaMM are present. Fig. 7 illustrates the network generated by these genes: each node in the network represents a gene, the node size is proportional to the number of pathways the specific gene belongs to, whereas edges between nodes indicate linked pathways. As it can be observed, few genes located in the centre of chart are the key genes linking all the remaining genes.

These genes and the pathways they belong to are listed in Table 2.

**Table 2:** Table lists the gene symbol and the KEGG pathway of selected genes

Gene symbol	Pathway name	Gene symbol	Pathway name
TNF	T cell receptor signaling pathway, osteoclast differentiation, natural killer cell mediated cytotoxicity, Fc epsilon RI signaling pathway, TGF-beta signaling pathway, amyotrophic lateral sclerosis (ALS), malaria	FYN	T cell receptor signaling pathway, osteoclast differentiation, natural killer cell mediated cytotoxicity, Fc epsilon RI signaling pathway, axon guidance, focal adhesion
PPP3CB	T cell receptor signaling pathway, osteoclast differentiation, natural killer cell mediated cytotoxicity, axon guidance, amyotrophic lateral sclerosis (ALS)	NFATC1	T cell receptor signaling pathway, osteoclast differentiation, natural killer cell mediated cytotoxicity, axon guidance
LCP2	T-cell receptor signaling pathway, osteoclast differentiation, natural Killer cell mediated cytoxicity, Fc epsilon RI signaling pathway	EGF	Pathway in cancer, focal adhesion, endometrial cancer
HGF	Pathway in cancer, malaria, focal adhesion	TGFB3	Pathway in cancer, malaria, TGF-beta signaling pathway
LAT	T-cell receptor signaling pathway, natural Killer cell mediated cytoxicity, Fc epsilon RI signaling pathway	CASP9	Pathways in cancer, amyotrophic lateral sclerosis (ALS), endometrial cancer
COMP	TGF-beta signaling pathway, focal adhesion, malaria	GAB2	Osteoclast differentiation, Fc epsilon RI signaling pathway
CRK	Pathway in cancer, focal adhesion	APC2	Pathway in cancer, endometrial cancer
BMP4	Pathway in cancer, TGF-beta signaling pathway	SPI1	Pathway in cancer, osteoclast differentiation
PDPK1	Focal adhesion, endometrial cancer	FCGR3B	Osteoclast differentiation, natural killer cell mediated cytoxicity
CD3E	T cell receptor signaling pathway	CD28	T cell receptor signaling pathway
CSF2RA	Pathways in cancer	SLIT2	Axon guidance
ID2	TGF-beta signaling pathway	E2F5	TGF-beta signaling pathway
PTPN11	Cytotoxicity mediated by killer cells	FZD2	Pathways in cancer
ACTN1	Focal adhesion	CFL1	Axon guide
RHOD	Axon guide	MAP3K5	Amyotrophic lateral sclerosis (ALS)

Only 14% of the total number of genes has been selected with the WebGestalt tool

In particular, it can be noted that:

- The TNF gene is shared in 7 pathways, it is a multifunctional pro-inflammatory cytokine and represents an index of fibroblast activity in the regulation of a broad spectrum of biological processes, including cell proliferation, differentiation, apoptosis, metabolic lipids and coagulation within the bone marrow [53, 54].
- The FYN gene and the PPP3CB gene are present in 6 and 5 of the selected pathways, respectively. In particular, the FYN gene is a member of the kinase family and plays a role in controlling cell growth [55]. The PPP3CB gene is a phosphatase and has a specific role in regulating T lymphocytes [56].
- NFATC1 and LCP2 genes are present in 4 of the selected pathways. The function of the protein encoded by the NFATC1 gene is the regulation of multiple cytokines and other regulatory molecules, including some interleukins (IL-2, IL-3, IL-4, IL-5, IL-6, IL-8, GM CSF), interferon (IFN)-$\nu ,$ tumor necrosis factor (TNF)-$\alpha ,$ CD40 ligand (CD154), and CD95 ligand (FasL). The NFATC1 protein is mainly studied in T cells (calcineurin-dependent 1) and is involved in the immune responses of lymphocytes B, NK cells, macrophages, mast cells, and eosinophils [20, 54, 57]. The LCP2 protein acts as a T cell substrate (TCR), therefore it plays a role in the transduction of the intracellular signal mediated by TCR [58].
- EGF, HGF, TGFB3, LAT, CASP9, and COMP genes are present in 3 of the 11 pathways, they encode for proteins involved in the growth, proliferation and differentiation of many cell types. In particular, EGF is a potent mitogen factor [59], while HGF binds to the hepatocyte growth factor receptor to regulate morphogenesis, growth and cellular motility and is secreted by mesenchymal cells. The HGF therefore acts as a multifunctional cytokine on predominantly epithelial cells and plays a role in angiogenesis, tumorigenesis, and tissue regeneration [60, 61]. TGFB3 protein is a ligand of the various TGF-beta receptors and leads to the recruitment and activation of SMAD family transcription factors and regulates a myriad of mainly immunosuppressive responses [18, 62–64]. The LAT protein forms, along with several adaptive proteins, a complex that creates a multimolecular signaling network located at the TCR engagement site. The role played by LAT protein underlines the complex modulation performed by fibroblasts of uncommon metaMM subsets in regulating immune response [65]. The CASP9 gene encodes for a protein whose function is comparable to a tumor suppressor and its functional polymorphisms may be responsible for alterations in proliferation [66]. The protein encoded by the gene COMP family belonging to the thrombospondin family carries out direct action on the mechanical integrity of the extracellular matrix, intervening in the interface between mineralized and non-mineralized regions [67, 68]. This protein is involved in the interaction between fibroblasts and osteoblasts, and is related to the significant changes caused by the nature of mechanical stress by the mineralized fibrocartilage to the bone [69].
- GAB2, CRK, APC2, BMP4, SPI1, PDPK1 and FCGR3B genes are only present in 2 of the 11 pathways selected, specifically GAB2 and CRK, are adapters for the transmission of various signals, GAB2 protein responds to receptors stimuli cytokines, growth factors, and antigen receptors [70] while CRK protein binds several tyrosine-phosphorylated proteins [71]. The APC2 gene encodes a protein that promotes the assembly of a multiproteic complex responsible for the control of beta-catenin cytoplasmic levels and is crucial in interaction with cytoskeletal proteins [72]. The BMP4 gene belongs to the superfamily of TGF-beta proteins [73] and the SPI1 gene encodes for a transcription factor that activates gene expression in the myeloid line and the development of B-lymphoid cells [74]. The PDPK1 gene encodes for a serine-threonine kinase, crucial to regulating cell migration. PDPK1 is a signal transducer for PI3K and activates multiple downstream effectors, it represents a focal point in the coordination of signals from the extracellular environment to the cytoskeleton PLC$\nu$ [75]. Finally, the FCGR3B gene encodes for a low affinity receptor for the Fc region of gamma immunoglobulins (IgG) and is capable of capturing immune complexes [76].
- Gene residues individually present in the 11 pathways, such as the CD3E gene and the CD28 gene may be involved in some cellular processes mainly related to an immune response [75]. The immune response is integrated with the CSF2RA [76] and SLIT2 genes [77]. A second set of genes promotes cell proliferation control; indeed, the ID2 gene belongs to transcriptional factor regulators and negatively regulates cell differentiation [78]. E2F5 genes and PTPN11 are important in cell cycle control and tumor suppressor genes in oncogenic transformation [79, 80]. The last group consists of FZD2 [81], ACTN1 [82], CFL1 [83] and RHOD [84, 85], these genes are involved in the organization of the cytoskeleton. Finally, MAPK gene encoding MAP3K5, responsible for the activation of several downstream effects, in particular transcription factors, which regulate different cellular responses [86]. The functional network created by the 30 genes in the 11 pathways selected and belonging to the uncommon subset of metaMM shows that fibroblasts have acquired additional properties from those belonging to the common metaMGUS subset that favor the “activated fibroblast” condition.

### Validation of post-processing procedure as preliminary step for functional analysis

It is important to note that the functional analysis of common genes, found through the post-processing procedure, is correlated with the information extracted studying the two clinical conditions at the same time. In fact, focusing on this aim, the NMF has been also performed on the matrix concatenating the MGUS and MM conditions with a rank equal to $$r=5.$$ This rank selection respects the theory of the rank of a concatenation matrix (that is $$rank([A_1,A_2])<rank(A_1)+rank(A_2),$$ where $$[A_1,A_2]$$ is the concatenation per column of the matrices $$A_1$$ and $$A_2).$$

**Fig. 8:**
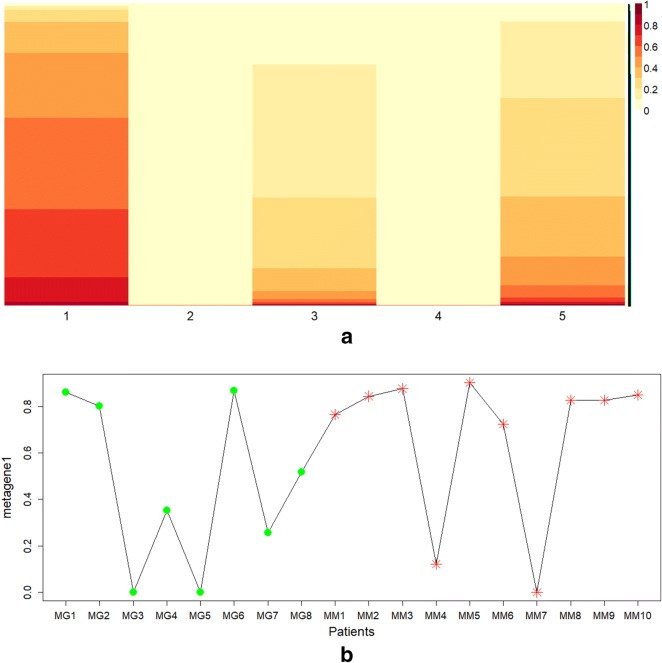
**a** Heatmap plot of basis matrix extracted from the gene expression profile data matrix. This matrix has been obtained concatenating expression data from 10 patients with MM condition and 8 patients with MGUS condition. Five metagenes were automatically extracted with a rank of the factorization = 5. Values in each column of the basis matrix have been normalized by row and sorted to show higher values on the bottom and lower values on the top of the heatmap. The first metagene was automatically identified as the most representative metagene of the whole dataset of the two conditions, since it presented the highest number of relevant values. **b** Density level of the coefficient matrix for each patient in the first metagene. Values in the coefficient matrix have been normalized by column to a clearer result representation. The MM and MGUS conditions are marked by circle and star markers, respectively. Higher values represent a greater influence of genes in the metagene on the corresponding patient

The heatmap plot of the reordered basis matrix, obtained from the NMF, shown in Fig. 8a, allows to identify the first column as the most informative metagene. From this metagene 1393 genes were identify through the extraction procedure; we refer to this subset as metaMGUSMM. It is worthy to note that metaMGUSMM contains a significant part of common genes previously extracted. Moreover, to further investigate the possible influence of the selected metagene among all patients, an analysis on the associated coefficient matrix was performed. Figure 8b plots the density level corresponding to the elements of the selected metagene in the coefficient matrix. The density values represent the weight of the selected metagene among patients who are distinguished by circle and star markers in accordance to the condition they belong to (circle corresponds to MGUS and star to MM, respectively). The higher is the value, the greater is the effect of the metagene over a specified condition; as it can be appreciated in the plot the majority corresponds to MM condition. This can be considered as an empirical evidence of the effectiveness of the functional analysis performed on the subset of genes as a tool to acquire an understanding on the activation of fibroblasts in bone marrow.

## Discussion

The combined use of NMF, gene extraction mechanism and post-processing procedure on fibroblast gene expression of patients with MGUS and MM, allows to select a reduced number of genes ideally involved in the activation of bone marrow fibroblasts (CAF) in the MM. Usually, as observed in solid tumors, a sub-population of CAF is studied on the basis of some expressed indicators, but the data are interpreted as true for all CAF populations. In fact, the term “cancer-associated fibroblasts” is misleading, since it groups these cells based solely on their position, despite the heterogeneity and roles in the different types of tumors. The common characteristic of CAF is the ability to alter the micro-environment and the behaviour of neoplastic cells. In fact, when a transformation is established in a cell, a symbiotic relationship that promotes tumor growth is generated with its micro-environment. This process is a crucial feature and those neoplastic cells that fail to develop this capacity will not overcome tumorigenic barriers and will remain quiescent (not evolution of the disease). In this study, we primarily sought to identify the specific genes associated with activation of the sub-population of CAF in the bone marrow micro-environment and aimed at understanding the role in the pathogenesis of MM. These genes, grouped into common and uncommon subsets compared to the two clinical conditions (MGUS and MM), were subsequently functionally analysed. The evaluation of the results obtained is purely descriptive and focused on 34 genes: 4 belonging to common genes and 30 to non-common genes in metaMGUS and metaMM. The 4 common genes show a fibroblast phenotype incapable of altering the bone marrow micro-environment. By contrast, the 30 genes belonging to the subset of uncommon genes of metaMGUS and metaMM have allowed fibroblasts to acquire additional properties other than those belonging to the subset of common metaMGUS and metaMM genes, that favour the “activated fibroblast” condition.

**Fig. 9:**
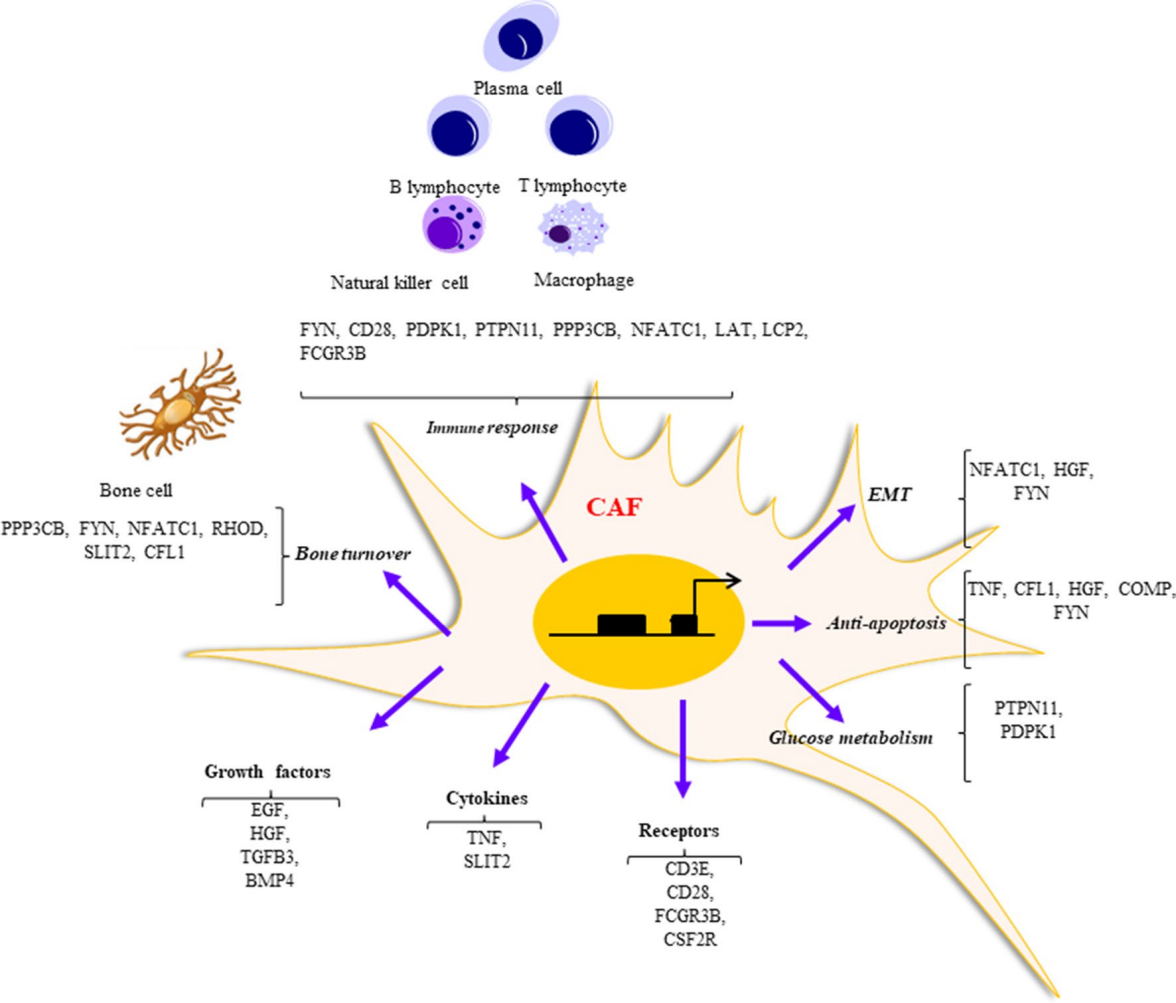
Functional network of different pathways genes. The functional network created by the genes in the 11 pathways selected and belonging to the subset of uncommon metaMM. The CAFs have acquired additional properties from those belonging to the common metaMGUS subset. In particular, the 30 selected genes regulate processes such as: epithelial–mesenchymal transition (EMT), immortalization, inhibition of apoptosis, changes in glucose metabolism and the mediation of the immune response. The phenotype acquired by fibroblasts shows properties that include homeostatic regulation and hormonal control of bone turnover (PPP3CB, FYN, NFATC1, RHOD, SLIT2, CFL1) and also mediate signals with specific receptors, growth factors and cytokines

In particular, as shown in Fig. 9, the functional pathways allowed to select proteins such as NFATC1, HGF and FYN proteins that favor epithelial–mesenchymal transition (EMT); add the immortalization processes linked to inhibition of apoptosis (TNF, CFL1, HGF, COMP, FYN) [53, 55, 87–89] and changes in glucose metabolism and the regulation of insulin signaling (PTPN11, PDPK1) [90]. The proteins encoded by the genes FYN, CD28, PDPK1, PTPN11 play a stimulating action of T cells [77, 91–93] while PPP3CB and NFATC1 regulate the signalling pathways of lymphocyte receptors B and T [56, 94]; as well as CD28, LAT, LCP2, FCGR3B are involved in mediating the immune response [58, 65, 76, 77]. In addition, the phenotype acquired by fibroblasts exhibits unique functional properties which also serve for osteoblastic differentiation. Indeed, in the evolution of MM disease, the action of the fibroblasts in the bone marrow niche is subject to a neuroendocrine regulation of bone metabolism. The fibers of the sympathetic nervous system (SNS), known to innervate the cortical bone, modulate a number of functions, including the homeostatic regulation and hormonal control of bone turnover [95]. In this regard, the gene products of PPP3CB, FYN, NFATC1, RHOD, SLIT2, CFL1 could be involved in the propagation of a vicious cycle characterized by the presence of osteolytic lesions, caused by prolonged declines in bone mineralization mediated by osteoblasts and increases bone resorption by osteoclasts. The signaling pathways are mediated by growth factors (EGF, HGF, TGFB3, BMP4), cytokines (TNF, SLIT2) and specific receptors (CD3E, CD28, FCGR3B, CSF2R), suggesting greater influence of activated fibroblasts in MM compared to the condition of MGUS. Therefore, in the progression of the disease, the selected 30 genes generate a clear interaction between activated fibroblasts and T lymphocytes, NK cells, osteoclasts, B lymphocytes, macrophages, and the same plasma cells. Thus, the subgroup of uncommon metaMGUS and metaMM genes may be considered candidates for determining the activation of bone marrow fibroblasts and actively involved in the progression of the disease from MGUS to MM. In addition, by adopting the style introduced for the different polarized immune cells, we could also subdivide the CAF into two functional subtypes: the F1 and F2 polarized fibroblasts [17, 18]. The subtype F1 could be associated with the MGUS condition because it is characterized by genes that alone are not related to the activation process of fibroblasts, although it is possible that they are able to work in conjunction with uncommon genes of metaMGUS and metaMM. The subtype F2, instead, characterized by the uncommon genes of metaMGUS and metaMM, can be associated with the MM condition because it is able to create an altered dialogue between the plasma cells and the bone marrow. Although some of these genes have already been validated by *in vitro* and *in vivo* experiments, as the literature indicates, the rest will be the object of our attention in the near future.

## Conclusion

In conclusion, we applied a peculiar methodology based on NMF method to gene expression profiles of fibroblasts from patients with MGUS and MM to identify—in an automatic manner—a limited number of genes which are possible candidates associated with activation of fibroblasts in the bone marrow in patients with MM compared to MGUS. We recognize that these in vitro results almost certainly represent an incomplete representation of the normal fibroblast response in the bone marrow microenvironment during the progression of the disease from MGUS to MM, for experimental times. However, we believe that the emerged picture strongly suggests a very broad role for fibroblast in the orchestration of this important process. Furthermore, it should be noted that only with a greater understanding of the relationship between plasma cells and stromal bone marrow cells can we have suggestions for a more targeted therapy and these results provide a further contribution. In fact, a pharmacological treatment with a simultaneous action against the genes in the selected pathways, rather than on the single gene, could have a greater effect on the evolution of the disease. Therefore, the development of combinatorial therapies for both CAF and neoplastic cells could be promising in clinical practice.

## Additional file

**Additional file 1: Tables S1 and S2.** Genes and their corresponding median value of MGUS and MM.

## Abbreviations

MM: multiple myeloma
NMF: nonnegative matrix factorization
PCA: principal component analysis
MGUS: monoclonal gammopathy of undetermined significance
PC: plasma cells
HSC: hematopoietic stem cell
BMSCs: stem cells of the bone mesenchymal
CAFs: activated fibroblasts cells
FCS: fetal calf serum
RPMI: Rosewell Park Memorial Institute 1640 Medium
EDTA: ethylendiaminotetraacetate
PBS: phosphate-buffered saline
SFM: FCS-free medium
LOESS: local regression
WebGestalt: WEBbased GEne SeT AnaLysis Toolket
Prok-1: 1 prokineticin precursor
GNRHR: gonadotropin releasing hormone 1 leutinizing-releasing hormone
AHSG: alpha-2-HSglycoprotein
b2-M: beta-2-microglobulin
S1P: Sphingosine 1-phosphate
TCR: T cell substrate
MoAb: monoclonal antibody
IgG: gamma immunoglobulins
EMT: mesenchymal transition
SNS: sympathetic nervous system

## Footnotes

1 https://www.ncbi.nlm.nih.gov/geo/query/acc.cgi?acc=GSE24990.

2 https://www.ncbi.nlm.nih.gov/geo/query/acc.cgi?acc=GPL2136.

3 Heatmap tool is frequently adopted to illustrate trends in genetic data which are not clearly visible using other visualization techniques. This plot associates a color to each numerical values in a matrix so that the relevance of a row element in a column can be easily highlighted.

4 This divergence is frequently adopted in microarray data analysis since it corresponds to the maximum likelihood estimation under independent Poisson assumption. [39]

5 WebGestalt is a functional enrichment analysis web tool that using statistics is able to translate gene lists into biological insights [44].
