# Supplementary material for: Improving knowledge on the activation of bone marrow fibroblasts in MGUS and MM disease through the automatic extraction of genes via a nonnegative matrix factorization approach on gene expression profiles
Source: J Transl Med. 2018 Aug 3;16:217. doi: 10.1186/s12967-018-1589-1 (PMC6076394; doi:10.1186/s12967-018-1589-1)
Supplement: Supplementary file 1 — Additional file 1: Tables S1 and S2. Genes and their corresponding median value of MGUS and MM. [file 12967_2018_1589_MOESM1_ESM.pdf]

Table S1- Common genes selected from metaMGUS and metaMM. The columns report the gene symbol, the description and the median values of MGUS and MM, respectively. In particular, the median values correspond to the median values of gene expression in all the analysed samples from 8 patients for MGUS and 10 patients for MM.

Gene symbol	Description	Median values of MM	Median values of MGUS
**B2M**	Beta-2-microglobulin	0.803665	1.138496
**GON4L**	Hypothetical protein FLJ23040	1.025625	0.705567
**Trappc11**	Hypothetical protein FLJ12716	0.953861	0.664985
**WHSC1**	Homo Sapiens clone B3B3E13 chromosome 4p16.3 DNA fragment	1.444996	1.208297
**MMP24**	Matrix metalloproteinase 24 membrane-inserted	1.399972	1.638051
**AHSG**	Alpha-2-HS-glycoprotein	0.590326	0.661648
**RGS16**	Regulator of G-protein signalling 16	0.665892	0.533122
**ZFP64**	Zinc finger protein 338	0.549547	0.632147
**IGSF3**	Immunoglobulin superfamily. member 3	1.309410	1.137218
**KRT37**	Keratin. hair. acidic. 7	0.947509	0.691861
**PROK1**	Prokineticin 1 precursor	0.612389	0.597899
**OR5G1P**	Olfactory receptor. family 5. subfamily G. member 1 pseudogene	0.740104	0.901389
**HYAL3**	Hyaluronoglucosaminidase 3	0.905676	1.216750
**RIG-like 14-1 (LOC51047)**	RIG-like 14-1	0.957418	0.843811
**FSCN2**	Fascin homolog 2. actin-bundling protein. retinal Strongylocentrotus purpuratus	1.555132	1.301086
**KANK2**	Hypothetical protein FLJ20004	1.095215	0.642862
**TVP23B**	CGI-148 protein	0.588956	0.092288
**KLF5**	Kruppel-like factor 5 intestinal	0.667383	0.687529
**LGR5**	G protein-coupled receptor 49	0.956719	0.833937
**GNRHR**	Gonadotropin-releasing hormone 1 leutinizing-releasing hormone	0.343268	0.162134
**SH3BP5L**	KIAA1720 protein	0.815569	0.785576

**ANXA11**	Annexin A11	0.999355	0.945053
**PRDM14**	PR domain containing 14	0.950124	0.733451
**C2orf42**	Hypothetical protein FLJ20558	1.009160	0.851449

Table S2- Uncommon genes between metaMGUS and metaMM found in metaMM. The columns in the table report the gene symbol, the description and the median values of MM and MGUS. In particular, the median values of MGUS and MM correspond to the median values of gene expression all samples analysed by 8 patients for MGUS and 10 patients for MM.

Gene symbol	Description	Median values of MM	Median values of MGUS
**APC2**	Adenomatosis polyposis coli	0.175976	0.167160
**SERPINB9**	Serine or cysteine proteinase inhibitor, clade B ovalbumin, member 9	0.880388	1.013527
**VPS54**	Tumor antigen SLP-8p	0.193500	0.222565
**LOC101929104**	Mitochondrial import receptor Tom22	0.882351	0.733234
**ETV1**	Ets variant gene 3	0.854903	1.275559
**ANKFY1**	ANKHZN protein	0.969263	0.697040
**CTSV**	Cathepsin L2	1.472927	1.303946
**CASK**	Trinucleotide repeat containing 8	0.317374	0.297790
**SELT**	Selenoprotein T	0.523566	0.804447
**ARID1A**	Uncharacterized bone marrow protein BM029	0.788157	0.889967
**AK3**	Adenylate kinase 3 alpha like	1.872808	1.401019
**ST18**	KIAA0535 gene product	1.262914	0.893420
**CFL1**	Cofilin 1 non-muscle	1.978321	1.397946
**CEP104**	KIAA0562 gene product	1.126382	1.056969
**OMG**	Oligodendrocyte myelin glycoprotein	0.744629	0.607513
**CRK**	V-crk sarcoma virus CT10 oncogene homolog avian -like	0.642147	0.873722
**ZNF24**	Zinc finger protein 24 KOX 17	1.011721	0.942563
**NUDT1**	Nudix nucleoside diphosphate linked moiety X -type motif 7	1.227940	1.276103
**SNW1**	SKI-interacting protein	0.485091	0.687023
**OR2S2**	Olfactory receptor. family 2. subfamily S. member 2	0.785907	1.131711
**ZNF14**	Zinc finger protein 14 KOX 6	0.606624	0.519972
**GPN3**	Protein x 0004	1.254619	1.246093
**CCL11**	Small inducible cytokine subfamily A Cys-Cys . member 11 eotaxin	0.682041	0.376786
**ZCCHC17**	Human putative ribosomal protein S1 mRNA	0.980212	0.853457

**B4GALT4**	UDP-Gal:betaGlcNAc beta 1.4-galactosyltransferase. polypeptide 4	1.281967	1.325816
**NME8**	NM23-H8	1.219057	0.747027
**CD3E**	CD3E antigen. epsilon polypeptide TiT3 complex	1.024422	1.384596
**FCN1**	Ficolin collagen fibrinogen domain containing 1	1.067468	0.770358
**ZSCAN12**	KIAA0426 gene product	0.299564	0.381118
**DARS**	Aspartyl-tRNA synthetase	1.088727	1.667536
**SLC26A4**	Solute carrier family 26. member 4	0.561984	0.588722
**NFATC1**	Nuclear factor of activated T-cells. cytoplasmic. calcineurin-dependent 3	0.572047	0.541798
**EDNRA**	Endothelin receptor type A	1.168090	0.967136
**PTPN11**	Protein tyrosine phosphatase. non-receptor type 11	0.765553	0.703333
**GALR3**	Galanin receptor 3	1.057470	0.899316
**DDX4**	VASA protein	0.393849	0.443761
**USP28**	Ubiquitin specific protease 28	0.374006	0.646873
**RRNAD1**	CGI-41 protein	0.879972	0.851924
**MRPS5**	Mitochondrial ribosomal protein S5	1.112703	1.141840
**GALNT6**	UDP-N-acetyl-alpha-D-galactosamine:polypeptide N-acetylgalactosaminyltransferase 6 GalNAc-T6	0.530203	0.804280
**USP38**	HP43.8KD protein	0.314880	0.333407
**Chrnb4**	Cholinergic receptor. nicotinic. beta polypeptide 4	1.043551	0.804825
**STS**	Human DNA sequence from PAC 30P20 on chromosome Xq21.1-Xq21.3. Contains set pseudogene. ESTs and STS	0.964794	1.026304
**DDOST**	Dolichyl-diphosphooligosaccharide-protein glycosyltransferase	0.338263	0.342192
**SPI1**	Spleen focus forming virus SFFV proviral integration oncogene spi1	1.139172	1.777054
**PDSS1**	Trans-prenyltransferase	0.872644	1.370615
**SRGN**	Proteoglycan 1. secretory granule	1.379783	0.871478
**PDPK1**	3-phosphoinositide dependent protein kinase1	0.529942	0.429391
**ID2**	Inhibitor of DNA binding 1. dominant negative helix-loop-helix protein	0.783597	0.723752
**KCNA4**	Potassium voltage-gated channel. shaker-related subfamily. member 4	1.003285	1.116546

**PICALM**	Phosphatidylinositol binding clathrin assembly protein	1.448186	1.030968
**SLIT2**	Slit homolog 2 Drosophila	1.347214	1.098022
**ENPP2**	Ectonucleotide pyrophosphatase phosphodiesterase 2 autotaxin	1.692701	1.233976
**CDS1**	CDP-diacylglycerol synthase phosphatidate cytidylyltransferase 1	1.357250	1.201198
**FXYD7**	ESTs	0.876743	0.880739
**ULK2**	Unc-51-like kinase 2 C. elegans	0.350618	0.323723
**MAGEH1**	APR-1 protein	1.351134	1.091539
**TM9SF4**	KIAA0255 gene product	0.776017	0.808375
**PCAT1**	Prostate cancer associated protein 1	1.016242	0.803037
**FER1L4**	Fer-1-like 4 C. elegans	1.070324	1.179898
**PTTG1**	Fibroblast growth factor 2 basic	0.373790	0.438797
**ANKRD26**	KIAA1074 protein	0.638264	1.343675
**KCNH3**	Potassium voltage-gated channel. subfamily H eag-related . member 3	0.918936	0.868978
**GPR52**	G protein-coupled receptor 52	1.552585	2.100040
**CPVL**	Carboxypeptidase. vitellogenic-like	0.934009	1.496231
**TGFB3**	Transforming growth factor. beta 3	0.740539	0.710438
**ADCY10**	Soluble adenylyl cyclase	1.002955	0.658123
**ZNF286A**	Peroxisome proliferative activated receptor. alpha-like	0.798243	1.369890
**GAB2**	GRB2-associated binding protein 2	1.621051	1.703815
**MAP4K1**	Mitogen-activated protein kinase kinase kinase kinase 1	1.087872	0.940820
**DYNC1H1**	Dynein. cytoplasmic. heavy polypeptide 1	1.499002	1.251249
**ZFHX2**	KIAA1762 protein	0.927258	1.115033
**Txlna**	Hypothetical protein FLJ11209	0.519810	0.550158
**KRT15**	Keratin 15	1.152301	1.344766
**TBK1**	TANK-binding kinase 1	0.436002	0.557374
**RAB1C**	Putative GTP-binding protein similar to RAY RAB1C	0.399786	0.307254
**MRPL2**	Mitochondrial ribosomal protein L2	1.243286	1.803796

**SLC13A2**	Solute carrier family 13 sodium-dependent dicarboxylate transporter . member 2	1.070961	1.152523
**ZNF106**	Zinc finger protein 106	1.462370	1.904974
**TNF**	Tumor necrosis factor TNF superfamily. member 2	0.936268	0.830499
**MRPL42**	Mitochondrial ribosomal protein L42	1.312141	1.143862
**DNAJC30**	DNAJ domain-containing	1.117829	1.167788
**ELL**	ELL gene 11-19 lysine-rich leukemia gene	0.359967	0.46215
**RGS8**	Regulator of G-protein signalling 8	1.351951	1.133003
**NR1D2**	Nuclear receptor subfamily 1. group D. member 2	1.091516	1.012955
**CD28**	CD28 antigen Tp44	0.342348	0.235467
**ZNF664-FAM101A**	Hypothetical protein	0.631110	1.388829
**COX5BP6**	Cytochrome c oxidase subunit Vb	0.789345	0.919977
**WISP1**	WNT1 inducible signaling pathway protein 1	0.883377	0.933203
**PPP3CB**	Protein phosphatase 3 formerly 2B . catalytic subunit. beta isoform calcineurin A beta	0.939975	0.634449
**TRA**	T cell receptor alpha locus	0.640758	0.443213
**LOC100133284**	Striatin. calmodulin binding protein	1.206089	1.474255
**ARNTL2**	Transcription factor BMAL2	1.278001	1.239264
**SNRPG**	Small nuclear ribonucleoprotein polypeptide G	1.183811	1.304376
**TNRC6A**	KIAA1460 protein	0.867007	1.160592
**FBXW7**	F-box and WD-40 domain protein 7 archipelago homolog. Drosophila	0.539158	1.013905
**SNRPF**	Sm protein F	1.298032	1.032848
**PREP**	Prolyl endopeptidase	0.332721	0.583155
**UBA1**	Ubiquitin-activating enzyme E1 A1S9T and BN75 temperature sensitivity complementing	0.356663	0.158528
**MBD6**	KIAA1887 protein	0.778144	0.240557
**BACE2**	Beta-site APP-cleaving enzyme	0.799644	1.233825
**DCUN1D1**	RP42 homolog	0.976125	0.921353
**SMIM3**	Integral membrane protein 3	0.924781	0.846095
**SDF2L1**	Stromal cell-derived factor 2-like 1	1.025839	1.345788
**ZNF678**	Hypothetical protein MGC15634	1.148892	1.070157

**BMP4**	Bone morphogenetic protein 4	1.087429	1.387971
**POGLUT1**	X 010 protein	0.572979	0.586705
**MORF4L2**	MORF-related gene X	0.698633	1.032204
**RRP15**	KIAA0507 protein	0.602829	0.384672
**LDHA**	Lactate dehydrogenase A	1.086640	1.628555
**GOLPH3L**	Hypothetical protein FLJ10687	1.363248	1.571834
**HGF**	Hepatocyte growth factor hepapoietin A; scatter factor	1.644962	1.689652
**TTTY7**	Testis transcript Y 7	0.787892	1.198582
**TIMM10B**	Fracture callus 1 homolog rat	0.862313	1.219299
**ZNF706**	HSPC038 protein	0.604956	0.749589
**RHOD**	Ras homolog gene family. member D	1.179139	1.670044
**EIF4A1**	Eukaryotic translation initiation factor 4A. isoform 2	1.190069	0.936028
**NLGN2**	Neuroligin	0.746668	0.614502
**QDPR**	Quinoid dihydropteridine reductase	0.972769	0.593320
**PMS2P1**	Postmeiotic segregation increased 2-like 1	0.806075	0.848609
**CDH18**	Cadherin 18. type 2	0.883477	1.343320
**WDR43**	KIAA0007 protein	0.882125	0.862392
**RIMBP2**	Peripheral benzodiazepine receptor-associated protein 1	0.943184	0.853214
**CLIC3**	Chloride channel 3	1.007021	1.078240
**DOCK7**	KIAA1771 protein	0.973177	0.895137
**SPINT3**	Serine protease inhibitor. Kunitz type. 3	0.550136	0.492881
**MAN1B1**	Mannosidase. alpha. class 1B. member 1	1.532104	1.516899
**CASP9**	Apoptotic protease activating factor	0.742047	1.125014
**PCF11**	PCF11p homolog	1.144863	0.822713
**NRXN1**	Neurexin 1	1.066146	1.033152
**AKR1B1**	Aldo-keto reductase family 1. member B1 aldose reductase	1.268493	1.238467
**LRP8**	Low density lipoprotein receptor-related protein 8. apolipoprotein e receptor	0.818569	0.708146
**NGLY1**	Hypothetical protein FLJ12409	0.776626	0.803186

**IRF5**	Interferon regulatory factor 5	1.020507	0.817257
**ENSA**	Endosulfine alpha	0.443276	0.885606
**DNAH5**	KIAA1603 protein	0.796842	0.723412
**S100A13**	S100 calcium binding protein A13	0.637607	0.739085
**SENP1**	Sentrin SUMO-specific protease	1.113717	1.466075
**EGF**	Cadherin. EGF LAG seven-pass G-type receptor 3 flamingo homolog. Drosophila	0.748213	0.978484
**MICALL1**	KIAA1668 protein	0.678081	0.387605
**MYF5**	Myogenic factor 5	1.580369	1.330095
**MYOZ2**	Myozenin 2	0.924871	1.068712
**BEGAIN**	KIAA1446 protein	1.131344	0.901690
**RNH1**	Ribonuclease angiogenin inhibitor	0.738804	0.719450
**OTC**	Ornithine carbamoyltransferase	1.685525	1.298201
**ANKLE2**	KIAA0692 protein	1.482089	1.912008
**FZD2**	Wingless-type MMTV integration site family. member 3A	1.033030	0.930118
**CAPN9**	Calpain 9 nCL-4	1.264316	1.106680
**RGP1**	KIAA0258 gene product	0.753138	0.473198
**AZGP1**	Alpha-2-glycoprotein 1. zinc	0.787439	0.924746
**CLCP2**	KIAA1802 protein	0.871565	1.093127
**HAPLN1**	Cartilage linking protein 1	0.761792	0.824901
**ASGR1**	Asialoglycoprotein receptor 1	1.013404	1.156353
**PNPLA8**	Intracellular membrane-associated calcium-independent phospholipase A2 gamma	0.993022	0.753920
**LCP2**	Lymphocyte cytosolic protein 2 SH2 domain-containing leukocyte protein of 76kD	0.756877	0.910425
**ADSL**	Adenylosuccinate lyase	0.513571	0.701675
**TATDN1**	CDA11 protein	0.782098	0.811012
**KCNQ2**	Potassium voltage-gated channel. KQT-like subfamily. member 2	1.064119	1.169697
**ATP10D**	ATPase. Class V. type 10D	0.828088	0.602347
**APIP**	CGI-29 protein	0.779293	0.784027
**STRN4**	Zinedin	1.378840	1.331451

**HOXD4**	Homeo box D4	1.759350	1.106071
**KLK13**	Kallikrein 13	0.708197	0.923935
**CSF2RA**	Colony stimulating factor 2 receptor. alpha. low-affinity granulocyte-macrophage	1.190615	0.904580
**H1FX**	H1 histone family. member X	1.527574	1.158693
**TIMM10**	Mitochondrial carrier family protein	0.899489	1.055313
**CDC45**	CDC45 cell division cycle 45-like S. cerevisiae	0.794961	0.754727
**NRBP1**	Nuclear receptor binding protein	1.039743	0.838734
**MAP3K5**	Mitogen-activated protein kinase kinase kinase 5	0.921271	1.056432
**LRP5**	Low density lipoprotein receptor-related protein 5	1.472782	1.131125
**PRNP**	Chromosome 20 open reading frame 30	0.407351	0.531181
**OSBPL3**	Oxysterol binding protein-like 3	0.919011	1.007554
**GAMT**	Guanidinoacetate N-methyltransferase	0.826861	1.198469
**HNRNPA1**	Heterogeneous nuclear ribonucleoprotein A1	0.654013	0.592714
**CCSER2**	KIAA1128 protein	1.172330	1.375814
**HDAC6**	Histone deacetylase 6	1.047143	1.307820
**ACTR2**	ARP2 actin-related protein 2 homolog yeast	1.778721	2.773088
**COMP**	Homo sapiens cDNA FLJ10183 fis. clone HEMBA1004276. highly similar to Homo sapiens AP-4 adaptor comp	0.860395	0.898790
**EIF2A**	CDA02 protein	0.776017	0.684588
**PIPSL**	Proteasome prosome. macropain 26S subunit. non-ATPase. 10	0.733711	0.886086
**FCGR3B**	Fc fragment of IgG. low affinity IIIb. receptor for CD16	1.536358	1.468015
**AHNAK**	AHNAK nucleoprotein desmoyokin	1.695618	1.594387
**CBX6**	Chromobox homolog 6	0.900156	0.735430
**E2F5**	Tumor differentially expressed 1	1.259257	1.326932
**LAT**	Linker for activation of T cells	0.688736	0.628996
**NDUFC1**	NADH dehydrogenase ubiquinone 1. subcomplex unknown. 1 6kD. KFYI	1.006807	0.814607
**CREB5**	CAMP response element-binding protein CRE-BPa	1.545728	1.042755
**IL2RB**	Interleukin 2 receptor. beta	0.723289	1.067273

**RPS10P5**	Ribosomal protein S10-like	0.565673	0.653710
**STKLD1**	Serine threonine kinase 9	0.555104	0.385034
**HYDIN**	KIAA1864 protein	0.931048	0.689630
**ATP10B**	Hypothetical protein FLJ21477	1.043590	0.918895
**ADRA2B**	Adrenergic. alpha-2B-. receptor	0.858683	1.178778
**XPR1**	Xenotropic and polytropic retrovirus receptor	0.779829	0.830146
**BTC**	Betacellulin	1.331437	1.009758
**TSPAN3**	Tetraspan 3	0.657112	0.391867
**CSNK1G3**	Casein kinase 1. gamma 3	0.905822	1.326315
**SNHG12**	PNAS-123	0.653745	0.541717
**ANKRA2**	Ankyrin repeat. family A RFXANK-like . 2	0.870570	0.880148
**ACTN1**	Actinin. alpha 1	0.542697	0.496318
**NASP**	Nuclear autoantigenic sperm protein histone-binding	1.075783	1.054038
**L3MBTL1**	H-l 3 mbt-like protein	0.954779	0.837488
**GUCY1A2**	Guanylate cyclase 1. soluble. alpha 2	0.535890	0.421897
**LGALS13**	Placental protein 13	0.800037	0.923168
**FYN**	FYN oncogene related to SRC. FGR. YES	0.845066	1.077178
**CCT7**	Chaperonin containing TCP1. subunit 7 eta	1.178323	1.302153
**SDF4**	Calcium binding protein Cab45 precursor	0.934956	0.987124
**NFYC**	Nuclear transcription factor Y. gamma	1.016095	1.344819
**CNR2**	Cannabinoid receptor 2 macrophage	1.435427	0.986083
**FAXDC2**	Fatty acid hydroxylase	0.983953	1.431886
**CWH43**	Hypothetical protein FLJ21511	1.276331	1.191748
**CBR3**	Carbonyl reductase 3	0.998912	1.130570
**FOXD1**	Forkhead box D1	0.816511	0.869880
**ZBTB22**	Zinc finger protein 297	0.724319	1.221150
**NRBF2**	Nuclear receptor binding factor-2	0.544962	0.595578
**GPM6B**	Glycoprotein M6B	1.184615	1.284501
